# Early chronotype with metabolic syndrome favours resting and exercise fat oxidation in relation to insulin‐stimulated non‐oxidative glucose disposal

**DOI:** 10.1113/EP090613

**Published:** 2022-09-19

**Authors:** Steven K. Malin, Mary‐Margaret E. Remchak, Anthony J. Smith, Tristan J. Ragland, Emily M. Heiston, Udeyvir Cheema

**Affiliations:** ^1^ Rutgers University New Brunswick NJ USA; ^2^ University of Virginia Charlottesville VA USA; ^3^ Division of Endocrinology Metabolism and Nutrition Rutgers University New Brunswick NJ USA; ^4^ New Jersey Institute for Food Nutrition and Health Rutgers University New Brunswick NJ USA; ^5^ Institute of Translational Medicine and Science Rutgers University New Brunswick NJ USA; ^6^ Virginia Commonwealth University Richmond VA USA

**Keywords:** exercise intensity, insulin sensitivity, metabolic syndrome, oxidative capacity, substrate utilization

## Abstract

**New Findings:**

- **What is the central question of this study?**
  Chronotype reflects differences in circadian‐mediated metabolic and hormonal profiles. But, does resting and/or exercise fuel use differ in early versus late chronotype as it relates to insulin sensitivity?
- **What are the main finding and its importance?**
  Early chronotypes with metabolic syndrome utilized more fat during rest and exercise independent of aerobic fitness when compared with late chronotypes. Early chronotypes were also more physically active throughout the day. Greater fat use was related to non‐oxidative glucose disposal. These findings suggest that early chronotypes have differences in fuel selection that associate with type 2 diabetes risk.

**Abstract:**

Early chronotypes (ECs) are often insulin‐sensitive, in part, due to physical activity behaviour. It is unclear, however, if chronotypes differ in resting and/or exercise fuel oxidation in relation to insulin action. Using the Morningness–Eveningness Questionnaire (MEQ), adults with metabolic syndrome (ATP III criteria) were classified as EC (MEQ = 63.7 ± 0.9, *n* = 24 (19F), 54.2 ± 1.2 years) or late chronotype (LC; MEQ = 47.2 ± 1.4, *n* = 27 (23F), 55.3 ± 1.5 years). Carbohydrate (CHO) and fat oxidation (FOX, indirect calorimetry) were determined at rest, 55% and 85% ${\dot{V}}_{O_{2}\max}$, along with heart rate and rating of perceived exertion. Physical activity patterns (accelerometers), body composition (DXA) and insulin sensitivity (clamp, 40 mU/m^2^/min, 90 mg/dl) with an indirect calorimetry for non‐oxidative glucose disposal (NOGD) were also determined. While demographics were similar, ECs had higher ${\dot{V}}_{O_{2}\max}$ (*P* = 0.02), NOGD (*P* < 0.001) and resting FOX (*P* = 0.02) than LCs. Both groups increased CHO reliance during exercise at 55% and 85% ${\dot{V}}_{O_{2}\max}$ (test effect, *P* < 0.01) from rest, although ECs used more fat (group effect, *P* < 0.01). ECs had lower sedentary behaviour and more physical activity during morning/midday (both, *P* < 0.05). FOX at 55% ${\dot{V}}_{O_{2}\max}$ correlated with ${\dot{V}}_{O_{2}\max}$ (*r* = 0.425, *P* = 0.004) whereas FOX at 85% ${\dot{V}}_{O_{2}\max}$ related to NOGD (*r* = 0.392, *P* = 0.022). ECs with metabolic syndrome used more fat in relation to insulin‐stimulated NOGD.

## 1 INTRODUCTION

Chronotype is a circadian classification identifying the preference of an individual to perform an activity or acknowledge alertness during different periods of the day. Early chronotypes (ECs) (i.e., preference to wake up early and/or perform activity earlier in the day) tend to have reduced prevalence of cardiovascular disease (CVD) risk including low triglycerides and C‐reactive protein, with high‐density lipoprotein (HDL) (Romero‐Cabrera et al., 2022). Furthermore, ECs tend to perform more overall physical activity (PA) than their late chronotype (LC) counterparts (Romero‐Cabrera et al., 2022; Ruddick‐Collins et al., 2018). The exact biological mechanism(s) by which chronotype confers higher disease risk is unknown, but insulin sensitivity is an important aetiological factor in the progression to type 2 diabetes and CVD (Haffner, 2003). Additionally, the inability to switch between lipid and carbohydrate fuel sources from fasted to fed states, known as metabolic inflexibility, may precede insulin resistance (Færch & Vaag, 2011; Goodpaster & Sparks, 2017).

Carbohydrate is considered the primary energy source during moderate‐to‐high‐intensity exercise in healthy controls, although training is known to lower reliance on glycogen/glucose flux and raise fat oxidation (Friedlander, Casazza, Horning, Buddinger, et al., 1998; Friedlander, et al., 2007). Somewhat discordant from fasting fat oxidation observations is that people with insulin resistance, prediabetes and/or type 2 diabetes have reduced muscle glycogen utilization and higher fat oxidation during exercise than matched healthy counterparts (Braun et al., 2004; Colberg et al., 1996; Goodpaster et al., 2002; Malin et al., 2013). Several factors have been purported to explain this apparent paradox (e.g., lipid oversupply and/or impaired oxidative capacity) (Braun et al., 2004; Colberg et al., 1996; Goodpaster et al., 2002; Malin et al., 2013), but no study to date has examined the impact of chronotype on exercise fuel selection. This is biologically relevant as circadian rhythm is a critical feature in chronic disease risk, in part, through energy metabolism disturbances (Poggiogalle et al., 2018; Zee et al., 2013). In fact, we recently reported that people classified as EC are more insulin‐sensitive in relation to insulin‐stimulated carbohydrate utilization, a marker of metabolic flexibility (Remchak et al., 2022). Moreover, we also demonstrated that ECs with metabolic syndrome were categorized as having lower TCA cycle intermediates as well as higher acyl‐carnitines, which was interpreted as supporting the hypothesis of mitochondrial disturbances in those with LCs (Remchak et al., 2022). It remains unknown, however, if fuel selection patterns differ from rest to moderate‐ or high‐intensity exercise in adults based on chronotype. Furthermore, the characterization of PA across chronotypes is often performed by questionnaire, with limited data via accelerometry to help depict potential fitness‐related differences. Therefore, we tested the hypothesis that ECs would use more fat during rest and transition to greater carbohydrate reliance during moderate‐ and high‐intensity exercise when compared with LCs. We also anticipated that greater resting fat oxidation as well as exercise metabolic flexibility (i.e., shift from rest to exercise fuel use) would associate with higher insulin sensitivity as measured by euglycaemic clamp and PA.

## 2 METHODS

### 2.1 Participants

Using the Morningness–Eveningness Questionnaire (MEQ) for this cross‐sectional study (Osonoi et al., 2014), 51 adults were classified as either EC (MEQ = 63.7 ± 0.9, *n* = 24 (19F), 54.2 ± 1.2 years) or LC (MEQ = 47.2 ± 1.4, *n* = 27 (23F), 55.3 ± 1.5 years). Specifically, participants were categorized as EC (i.e., MEQ scores ≥59) or LC (MEQ scores ≤58) to test if chronotype impacts resting and/or exercise fuel use (Osonoi et al., 2014). The Epworth Sleepiness Scale (https://www.cdc.gov/niosh/work‐hour‐training‐for‐nurses/02/epworth.pdf) was also used to assess the likelihood of nodding off or falling asleep during specific daily activities (i.e., watching TV, sitting inactive, in a car while stopped, etc.) to approximate sleep quality/duration as previously described (Crook et al., 2019). Participants were sedentary (i.e., <60 min/week of structured exercise), non‐smoking and free of CVD, cancer (within the last 5 years), respiratory complications, or known metabolic disease (e.g., type II diabetes, non‐alcoholic fatty liver disease, etc.). Participants were excluded if they took medication affecting substrate metabolism, blood flow or insulin sensitivity (e.g., metformin, angiotensin II receptor blockers). All participants underwent a resting/exercise electrocardiogram, clinical biochemistries and a medical exam by a study physician to ensure participant safety for exercise. This study is part of a larger clinical trial (Registration No. NCT03355469), and as such all people had metabolic syndrome as per ATP III guidelines. This included, after an overnight fast, blood collection to assess fasting glucose (fasting ≥100 mg/dl), high‐density lipoprotein (HDL) (women: <50 mg/dl; men: <40 mg/dl) and triglycerides (≥150 mg/dl). Waist circumference (women: ≥88 cm; men: ≥102 cm) was also measured up to three times using a plastic tape measure approximately 2 cm above the umbilicus and averaged. Blood pressure (systolic ≥130 mmHg and/or diastolic ≥85 mmHg) was lastly recorded after 5 min of rest in a seated position with feat positioned flat on the floor. A total of three blood pressure recordings occurred with 1–2 min rest in between and the data were averaged. All work conformed to the standards set by the latest version of the *Declaration of Helsinki*. Participants gave verbal and written informed consent before participating and the study was approved by our Institutional Review Board (IRB no. 19364 and no. Pro2020002029).

### 2.2 Body composition

Total body weight was assessed on a digital scale measured with participants wearing minimal clothing and without shoes. Waist circumference was measured in duplicate 2 cm above the umbilicus using a standard tape measure. Values within 0.5 cm were averaged. If a value was not within 0.5 cm, a third measure was recorded and averaged. Height was assessed with a stadiometer. Body mass index (BMI) was then calculated. Body fat, fat‐free mass (FFM), and visceral adipose tissue (VAT) were measured using dual‐energy X‐ray absorptiometry (DXA Horizon DXA System, Marlborough, MA, USA) following an appropriate 4‐h fast of food and beverage, including water, and abstaining from engaging in physical exercise for at least 4 h.

### 2.3 Maximal aerobic fitness and non‐exercise physical activity

Participants performed an incremental to maximal oxygen consumption test (${\dot{V}}_{O_{2}\max}$) on a treadmill. A self‐selected walking speed was maintained for the duration of the test. The incline was raised 2.5% every 2 min until volitional exhaustion. ${\dot{V}}_{O_{2}\max}$ was confirmed by at least three of the following four criteria: (1) a respiratory exchange ratio (RER) >1.1, (2) a plateau in ${\dot{V}}_{O_{2}}$ (<0.150 ml/min change), (3) heart rate (HR) achieved was within 10 beats of the age‐predicated HR max (220‐age), and/or (4) rating of perceived exertion (RPE) ≥17 a.u. on the Borg scale. Respiratory gases (i.e., ${\dot{V}}_{O_{2}}$ and ${\dot{V}}_{{\mathrm{CO}}_{2}}$) were collected throughout the test using indirect calorimetry (Vmax Encore, CareFusion, Yorba Linda, CA, USA). HR via electrocardiogram and RPE was recorded during the last 30 s of each stage. RPE in particular was assessed by investigators through the use of a 6–20 point scale, in which participants were asked to state/point to the level of exertion. ${\dot{V}}_{O_{2}\max}$ was determined as the highest ${\dot{V}}_{O_{2}}$ value achieved during the incremental test. Participants were also given a three‐axis accelerometer (Actigraph, Pensacola, FL, USA) and instructed to wear the device on the right hip. Participants were instructed to wear the device upon rising in the morning and remove the device before retiring to bed, for 7 days. A valid wear time for this measurement was considered a total of 4 days worn with an average of 10 h/day. Data included the mean wear time per day and the total number of days the device was worn. Freedson VM3 (’11) was used to determine the percentage of wear time spent in sedentary, light (LPA), moderate (MPA), or vigorous PA (VPA) per day (Freedson et al., 1998; Troiano et al., 2008). Based on recent work (Qian et al., 2021), groups were divided into morning (06.00–10.59 H), midday (11.00–14.59 h), and afternoon (15.00–19.00 h) to characterize how chronotype impacted sedentary and activity patterns across the day.

### 2.4 Metabolic control

Participants were instructed to refrain from strenuous non‐exercise PA as well as consumption of alcohol, caffeine and medications 24 h prior to the study visits. A low‐fat American Heart Association‐based diet consisting of 55% carbohydrates, 15% protein and 30% fat, with <10% from saturated fat, was provided to standardize diet prior to metabolic tests. Energy requirements for breakfast, lunch, dinner and snacks were determined via metabolic rate (RMR) tests from indirect calorimetry following an overnight fast and multiplied by a PA factor of 1.2.

### 2.5 Euglycaemic hyperinsulinaemic clamp

Participants arrived at the Clinical Research Unit between 06.00 and 08.00 h after an approximate 10–12 h overnight fast. A catheter was placed in the antecubital as well as dorsal hand or forearm vein for infusion and blood sampling, respectively. A primed (250 mU/m^2^/min) constant infusion (40 mU/m^2^/min) of insulin was administered via peristaltic infusion pumps (Harvard Apparatus, Holliston, MA, USA) for 120 min. Plasma glucose was collected every 5 min to determine the appropriate glucose infusion rate (GIR) to maintain a circulating glucose at 90 mg/dl (or about 5 mM). Plasma glucose samples were analysed immediately using the YSI 2300 StatPlus Glucose Analyzer system (Yellow Springs, OH, USA). Metabolic insulin sensitivity was defined as the GIR during the last 30 min of the clamp. Indirect calorimetry was also implemented during the steady‐state period for 15 min to capture respiratory gases. Non‐oxidative glucose disposal (NOGD) was then calculated as total carbohydrate oxidation minus GIR.

### 2.6 Resting and submaximal exercise fuel metabolism

On a separate day, after an overnight fast, participants reported to the laboratory between 07.00 and 09.00 h. Baseline respiratory gases and ventilation were collected for 5 min while standing quietly on the treadmill. Participants then completed two 15‐min bouts of exercise at 55% (moderate intensity) and 85% (high intensity) of their ${\dot{V}}_{O_{2}\max}$. Speed and grades were adjusted per individual to target metabolic loads. An approximate 2‐min break was provided after the first 15 min to foster successful completion. Respiratory gases were collected using indirect calorimetry during each 15‐min stage. During the last 30 s of each stage, HR via telemetry (Polar Electro, Kempele, Finland) and RPE were recorded. ${\dot{V}}_{O_{2}}$ and ${\dot{V}}_{{\mathrm{CO}}_{2}}$ were averaged over the last 2 min of each stage for the determination of fuel oxidation as performed before by our group (Gaitán et al., 2019). Calculations are shown here for convenience, including exercise metabolic flexibility:

1. Carbohydrate (CHO) oxidation rate (g/min) = 4.5850 ${\dot{V}}_{{\mathrm{CO}}_{2}}$ − 3.2255 ${\dot{V}}_{O_{2}}$
2. Fat oxidation rate (g/min) = 1.6946 ${\dot{V}}_{O_{2}}$ − 1.7012 ${\dot{V}}_{{\mathrm{CO}}_{2}}$
3. Energy expenditure = [(%CHO/100) × ${\dot{V}}_{O_{2}}$ × 5.05] + [(1 − %CHO/100) × ${\dot{V}}_{O_{2}}$ × 4.7]
4. Metabolic flexibility = difference between exercise and resting fuel oxidation

### 2.7 Statistical analysis

Data were analysed using SPSS Statistics (Version 24, IBM Corp., Armonk, NY, USA). An independent two‐tailed Student's *t‐*test was used to analyse baseline or rest group differences. A two‐ (group × test) or three‐way factor (group × test × time) repeated measures analysis of variance (ANOVA) was used when appropriate to determine group mean differences for outcomes. Given aerobic fitness was different between chronotype groups, we co‐varied for this on fat oxidation and NOGD in an effort to confirm chronotype effects. Pearson's correlation was used to examine associations. Data are means ± SD, and significance was accepted as *P* ≤ 0.05.

## 3 RESULTS

### 3.1 Participant characteristics

There were no significant statistical differences in age, body composition, or ATP III criteria between chronotypes (Table 1). However, ECs had lower HDL levels and higher ${\dot{V}}_{O_{2}\max}$ when scaled to FFM than LCs (*P* = 0.017; Table 1). Further, NOGD was significantly higher in ECs versus LCs (*P* = 0.001, without co‐varying) and remained significant after ${\dot{V}}_{O_{2}\max}$ and LPA adjustment (*P* = 0.002 and *P* = 0.03, respectively). Interestingly, in subgroup analysis (*n* = 31), ECs were more physically active as reflected by lower total sedentary behaviour in ECs versus LCs (72.3 ± 1.2 vs. 76.5 ± 1.6% *P* = 0.056). In fact, LCs became more sedentary across the day compared with ECs (group × time interaction; *P* = 0.041; Table 2). Moreover, ECs performed more activity during the morning (group effect, *P* = 0.054) and midday (11.00–14.59 h, *P* = 0.043) when compared with LCs (Table 2). There was no difference in VPA between groups.

**TABLE 1:** Participant characteristics

	Early	Late	*P*
Participant demographics			
N (M/F)	24 (5M/19F)	27 (4M/23F)	
Age (years)	54.2 ± 5.5	55.3 ± 7.8	0.570
Race/ethnicity			
African American	2	3	
Hispanic/Latino	2	0	
White	20	24	
MEQ score	63.7 ± 4.7	47.2 ± 6.3	<0.001
Epworth sleep score	6.8 ± 3.7	8.8 ± 4.3	0.104
Body composition			
Weight (kg)	95.7 ± 7.6	104.7 ± 3.8	0.064
BMI (kg/m^2^)	35.2 ± 2.6	37.0 ± 4.8	0.220
FFM (kg)	53.8 ± 2.2	54.6 ± 2.0	0.416
Body fat (%)	43.7 ± 3.3	45.2 ± 1.7	0.364
VAT volume (cm^2^)	932.5 ± 99.0	1025.0 ± 147.0	0.196
Aerobic fitness			
${\dot{V}}_{O_{2}\max}$ (l/min)	2.3 ± 0.2	2.2 ± 0.2	0.531
${\dot{V}}_{O_{2}\max}$ (ml/kg/min)	23.7 ± 2.6	21.3 ± 2.0	0.048
${\dot{V}}_{O_{2}\max}$ (ml/kg FFM/min)	42.8 ± 4.3	38.9 ± 3.5	0.025
Cardiometabolic disease risk			
ATP III score	3.4 ± 0.5	3.5 ± 0.9	0.559
WC (cm)	110.0 ± 9.7	113.9 ± 6.3	0.203
SBP (mmHg)	131.7 ± 14.9	134.2 ± 8.2	0.435
DBP (mmHg)	78.4 ± 10.9	79.0 ± 9.1	0.828
FPG (mg/dl)	95.8 ± 12.4	101.0 ± 6.7	0.131
TG (mg/dl)	135.8 ± 57.6	140.8 ± 47.7	0.707
HDL‐c (mg/dl)	43.6 ± 10.0	50.0 ± 9.7	0.017
GIR (mg/kg FFM/min)	4.8 ± 1.7	4.2 ± 0.4	0.359
NOGD (mg/kg FFM/min)	3.7 ± 1.5	1.4 ± 0.4	<0.001

Data are means ± SD and reflect the full data set available. Complete data present. Abbreviations: BMI, body mass index; DBP, diastolic blood pressure; FFM, fat‐free mass; FPG, fasting plasma glucose, 1 mM = 18 mg/dl; GIR, glucose infusion rate; HDL, high‐density lipoproteins, 1 mM = 38.67 mg/dl; NOGD, non‐oxidative glucose disposal; SBP, systolic blood pressure; TG, triglycerides, 1 mM = 88.5 mg/dl.

**TABLE 2:** Non‐exercise physical activity patterns

			*P*		
	Early	Late	Group effect	Test effect	Group by test effect
Total active wear time for day (%)					
Light physical activity	21.9 ± 1.9	19.3 ± 5.7	0.154	**<0.001**	0.500
Moderate physical activity	4.8 ± 2.4	4.1 ± 2.0			
Vigorous physical activity	0.06 ± 0.0	0.05 ± 0.0			
Total wear time in sedentary behaviour (%)					
Sedentary behaviour in morning	72.0 ± 9.8	68.2 ± 10.1	0.334	**0.001**	**0.041**
Sedentary behaviour in midday	69.2 ± 6.0	73.8 ± 9.1			
Sedentary behaviour in afternoon	73.1 ± 5.1	78.1 ± 5.8			
Total active wear time in morning (%)					
Light physical activity	21.3 ± 6.1	17.1 ± 8.5	**0.054**	**<0.001**	0.130
Moderate physical activity	5.2 ± 3.2	3.6 ± 1.0			
Vigorous physical activity	0.2 ± 0.1	0.1 ± 0.1			
Total active wear time in midday (%)					
Light physical activity	23.7 ± 3.6	19.5 ± 8.2	**0.043**	**<0.001**	0.114
Moderate physical activity	5.6 ± 4.1	3.6 ± 2.4			
Vigorous physical activity	0.03 ± 0.0	0.01 ± 0.0			
Total active wear time in afternoon (%)					
Light physical activity	20.4 ± 4.0	17.9 ± 5.8	0.131	**<0.001**	0.413
Moderate physical activity	3.7 ± 1.6	3.1 ± 2.0			
Vigorous physical activity	0.04 ± 0.0	0.01 ± 0.0			

Data are means ± SD. Subgroup analysis, early chronotype *n* = 13 of 24 versus late chronotype *n* = 18 of 27. Values shown in bold indicate statistical significance. Morning: 06.00–10.59 h; midday: 11.00–14.59 h; afternoon: 15.00–19.00 h.

### 3.2 Resting and submaximal exercise characteristics

HR, RPE, oxygen consumption and energy expenditure were not statistically different during rest, 55% or 85% ${\dot{V}}_{O_{2}\max}$ tests (Table 3).

**TABLE 3:** Submaximal exercise characteristics

			*P*		
	Early	Late	Group effect	Test effect	Group by test effect
HR (bpm)					
Rest	81.8 ± 8.5	81.0 ± 6.1	0.849	**<0.001**	0.925
55% ${\dot{V}}_{O_{2}\max}$	110.3 ± 0.4	112.4 ± 17.0			
85% ${\dot{V}}_{O_{2}\max}$	133.1 ± 7.7	133.4 ± 19.4			
RPE (a.u.)					
Rest	6.0 ± 0.0	6.2 ± 0.4	0.399	**<0.001**	0.928
55% ${\dot{V}}_{O_{2}\max}$	9.3 ± 1.7	9.8 ± 0.7			
85% ${\dot{V}}_{O_{2}\max}$	13.3 ± 1.0	13.5 ± 1.2			
VE (l/min)					
Rest	13.2 ± 3.3	13.5 ± 4.5	0.315	**<0.001**	0.666
55% ${\dot{V}}_{O_{2}\max}$	31.9 ± 2.3	30.7 ± 3.5			
85% ${\dot{V}}_{O_{2}\max}$	51.0 ± 4.9	50.9 ± 12.9			
${\dot{V}}_{O_{2}}$ (l/min)					
Rest	0.4 ± 0.1	0.4 ± 0.1	0.327	**<0.001**	0.671
55% ${\dot{V}}_{O_{2}\max}$	1.2 ± 0.1	1.2 ± 0.2			
85% ${\dot{V}}_{O_{2}\max}$	1.8 ± 0.2	1.7 ± 0.5			
${\dot{V}}_{O_{2}}$ (ml/kg FFM/min)					
Rest	8.9 ± 1.6	9.6 ± 0.3	0.117	**<0.001**	0.432
55% ${\dot{V}}_{O_{2}\max}$	30.9 ± 0.4	29.9 ± 0.9			
85% ${\dot{V}}_{O_{2}\max}$	45.6 ± 1.4	41.7 ± 3.7			
% ${\dot{V}}_{O_{2}\max}$					
Rest	16.6 ± 1.8	16.4 ± 3.5	0.126	**<0.001**	0.543
55% ${\dot{V}}_{O_{2}\max}$	53.5 ± 1.2	56.4 ± 7.1			
85% ${\dot{V}}_{O_{2}\max}$	80.3 ± 7.0	79.5 ± 6.6			
EE (kcal/min)					
Rest	1.8 ± 0.7	1.8 ± 0.6	0.646	**<0.001**	0.284
55% ${\dot{V}}_{O_{2}\max}$	5.9 ± 0.6	5.8 ± 0.9			
85% ${\dot{V}}_{O_{2}\max}$	8.8 ± 0.8	8.7 ± 2.7			

Data are means ± SD and reflect the full data set available. Values shown in bold indicate statistical significance. Abbreviations: EE, energy expenditure; FFM: fat‐free mass; HR, heart rate; RPE, rating of perceived exertion; VE, ventilation; ${\dot{V}}_{O_{2}}$, volume of oxygen.

### 3.3 Resting and submaximal fuel selection

**FIGURE 1:**
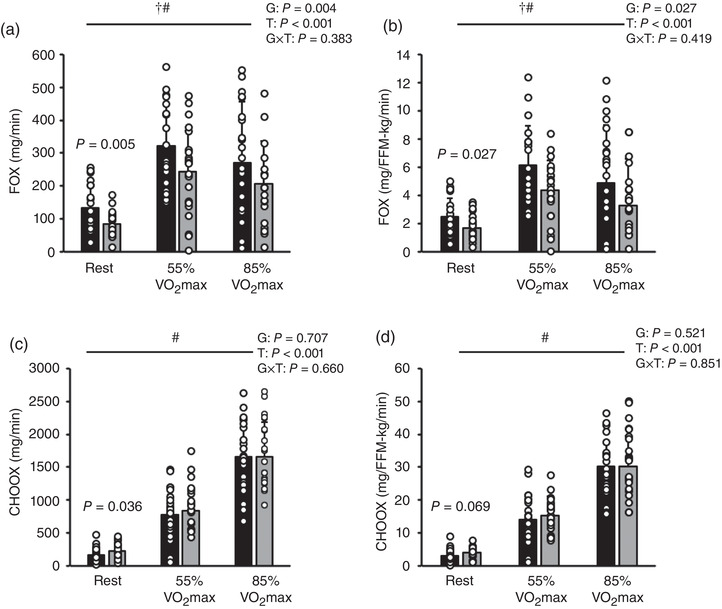
Substrate utilization during rest and submaximal exercise. Data are means ± SD and reflect the full data set available. (a, b) Absolute (a) and relative (b) fat oxidation (FOX). (c, d) Absolute (c) and relative (d) carbohydrate oxidation (CHOOX). ^†^Significant main effect of group; ^#^significant main effect of the test. Black bar, early chronotype; grey bar, late chronotype

Resting fat oxidation was higher in ECs compared with LCs independent of FFM (*t*‐test, *P* = 0.021, Figure 1). While both groups increased CHO oxidation during exercise at 55% and 85% ${\dot{V}}_{O_{2}\max}$ in an intensity‐based manner (intensity effect, *P* = 0.029, Figure 1), ECs maintained greater fat oxidation across all conditions (group effect, *P* = 0.029, Figure 1). Fat oxidation at rest and during exercise remained significant after co‐varying for ${\dot{V}}_{O_{2}\max}$ and LPA adjustment (group effect, *P* = 0.017 and *P* = 0.009, respectively). Nonetheless, there were no statistical differences in metabolic flexibility between groups at either 55% or 85% ${\dot{V}}_{O_{2}\max}$. Indeed, CHO oxidation increased similarly for ECs and LCs at 55% (11.9 ± 1.5 vs. 11.3 ± 1.1 mg/kg FFM/min, *P* = 0.747) and 85% ${\dot{V}}_{O_{2}\max}$ (27.8 ± 1.8 vs. 26.8 ± 1.9 mg/kg FFM/min, *P* = 0.718), while fat oxidation also showed elevations at 55% (3.3 ± 0.5 vs. 2.6 ± 0.3 mg/kg FFM/min, *P* = 0.260) and 85% ${\dot{V}}_{O_{2}\max}$ (2.4 ± 0.9 vs. 1.6 ± 0.5 mg/kg FFM/min, *P* = 0.428).

### 3.4 Correlations

**FIGURE 2:**
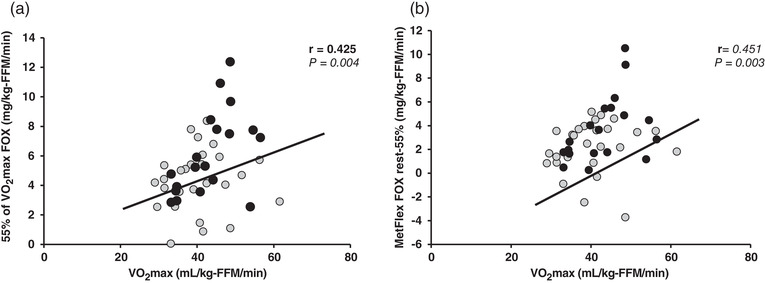
Correlation between changes in fat oxidation (FOX) and metabolic flexibility of fat (MetFlex) with ${\dot{V}}_{O_{2}\max}$. *n* = 24 for early and *n* = 27 for late chronotype. Black circle, early chronotype; grey circle, late chronotype

${\dot{V}}_{O_{2}\max}$ correlated with exercise fat oxidation (*r* = 0.425, *P* = 0.004) and metabolic flexibility at 55% ${\dot{V}}_{O_{2}\max}$ (*r* = 0.451, *P* = 0.003) (Figure 2). BMI was correlated with total sedentary behaviour (*r* = 0.397, *P* = 0.036), with afternoon sedentary behaviour specifically reaching statistical significance (*r* = 0.393, *P* = 0.039). Moreover, total LPA correlated with body weight (*r* = −0.413, *P* = 0.036) as well as metabolic insulin sensitivity (*r* = 0.42, *P* = 0.035). Although fat oxidation per se did not correlate with metabolic insulin sensitivity at rest, 55 or 85% ${\dot{V}}_{O_{2}\max}$ (data not shown), it is worth noting that fat oxidation at 85% ${\dot{V}}_{O_{2}\max}$ correlated with NOGD (*r* = 0.392, *P* = 0.022).

## 4 DISCUSSION

The principal finding of this study is that fasting and exercise fat oxidation are higher in early compared with later chronotypes with metabolic syndrome, independent of fitness and LPA. Interestingly, these findings were also independent of HR, RPE, oxygen consumption and energy expenditure during exercise. This latter observation highlights that workload does not explain differences between chronotypes and that our results are not mediated by sympathetic nervous system activity on fat mobilization and/or hepatic glucose production (Friedlander, Casazza, Horning, Huie, et al., 1998). Although these results are consistent with suggestions of low fasting fat oxidation in people with obesity as well as type 2 diabetes (Holloway et al., 2009; Houmard, 2008), it is important to highlight that LCs shifted fuel preference towards CHO oxidation during exercise in an equivalent manner to ECs. This suggests that exercise metabolic flexibility was present regardless of ECs using more fat. In addition, ECs engaged in less sedentary behaviour throughout the day and performed more PA in the morning and midday than LCs. These observations may have public health significance as more LPA paralleled higher ${\dot{V}}_{O_{2}\max}$ in ECs as well as being directly correlated with elevated metabolic insulin sensitivity. Thus, individual preference for engaging in activity during the earlier parts of the day may favourably impact metabolic health.

The reason ECs with metabolic syndrome utilized more fat during the fasting and/or exercise state could be due to several factors. Fat oxidation has been linked to skeletal muscle oxidative capacity (Bruce et al., 2003; Venables & Jeukendrup, 2008). Given that aerobic fitness is, at least partially, mediated by mitochondrial capacity, it would be fair to anticipate better fat oxidation in ECs if they had higher ${\dot{V}}_{O_{2}\max}$. Interestingly, higher aerobic fitness was directly correlated with exercise fat oxidation and metabolic flexibility at 55% ${\dot{V}}_{O_{2}\max}$. Fat oxidation was scaled to FFM, suggesting that muscle mass per se is not likely a factor explaining differences between chronotypes. Instead, the fitness association to fat oxidation more likely relates to the quality of muscle within ECs. The present findings are in line with prior work by our group highlighting that LCs have elevations in blood lactate and TCA cycle intermediates when compared with ECs that parallel lower metabolic insulin sensitivity (Remchak et al., 2022). Somewhat surprisingly, though, we report no direct correlation of higher fat oxidation with metabolic insulin sensitivity herein. Instead, fat oxidation at 85% ${\dot{V}}_{O_{2}\max}$ correlated with NOGD. This suggests that mechanisms related to higher fat oxidation during more intense activity may be related to insulin‐stimulated glycogen storage. Therefore, it might be speculated that increases in fat utilization at higher intensities ‘rescue’ the cell from lipid abnormalities (e.g., ceramides/diacylglycerol/incomplete lipids, etc.) related to insulin resistance (Goodpaster & Coen, 2012). Interestingly, it is worth noting that lipid abnormalities regarding low HDL were not seen in our cohort of LCs as previously described in evening adults (Romero‐Cabrera et al., 2022). In fact, HDL was higher in those with LC in our study when compared with ECs. We did not design the study to specifically discern cholesterol kinetics, but a possibility is that our LCs are a mix of intermediate and evening people as reflected by MEQ score averages when compared with prior work that split people into classifications of morning, intermediate and evening (Romero‐Cabrera et al., 2022). In either case, future work using stable isotopes and/or biopsies is required to confirm the location of fat metabolism disturbance to improve precision care by chronotype particularly since fat oxidation remained significant between groups after co‐varying for fitness and LPA.

Another reason chronotype may impact fuel use relates to adipose tissue accretion (Goodpaster et al., 2002). Exercise stimulates lipolytic activity in visceral fat tissue to a greater extent than gluteofemoral fat mass (Carey et al., 1996; Kanaley et al., 1993). If individuals with LC had less visceral fat than adults with EC, it may restrain plasma‐free fatty acid availability and subsequent rise in whole‐body fat utilization. Although we did not measure lipolysis via tracers in this study, we found no differences in BMI, waist circumference, VAT or body‐fat percentage between groups. Moreover, we observed no direct relation of body composition with fat oxidation in this study, despite LCs engaging in more sedentary behaviour and this relating to body weight and BMI – a finding consistent with the literature (Henson et al., 2020; Romero‐Cabrera et al., 2022; Shechter & St‐Onge, 2014). Thus, adiposity is unlikely to explain our results in fuel metabolism.

The capacity to increase CHO reliance during moderate‐ and high‐intensity exercise to a similar extent in people with LC versus EC with metabolic syndrome is noteworthy. This rise in CHO oxidation may be either a direct result of elevated CHO oxidation, or a compensatory rise due to decreased fat oxidation. We did not incorporate stable isotopes in this study to discern glucose flux, nor did we determine substrate levels during exercise to assess these possibilities. However, it is possible that LCs had elevated rates of hepatic glucose production that promoted blood glucose availability for increased oxidation in the fasted state. Prior work we conducted showcased that the gluconeogenic precursors lactate and alanine were elevated in people categorized as LC (Remchak et al., 2022). Interestingly, however, we report here no differences in fasting blood glucose, suggesting that despite possible elevations in gluconeogenic precursors, there was a matching of glucose disposal in the fasted state. While consistent with greater CHO oxidation, the idea that individuals with EC and LC rely on CHO to a similar extent during exercise highlights that exercise is capable of increasing CHO flux regardless of fasting metabolism differences. Moreover, when compared with our prior work showing that insulin does not promote CHO utilization in LCs versus ECs, these present findings highlight that exercise is capable of regulating blood glucose during muscle contraction in LCs independent of insulin action (Braun et al., 2004; Colberg et al., 1996; Goodpaster et al., 2002; Malin et al., 2013). Together, the mass action effects of glucose per se are unlikely to affect glucose uptake during exercise in LCs (Colberg et al., 1996). In contrast, muscle glycogen use during exercise is related to resting glycogen concentrations (Sherman et al., 1981). If our participants with LC began exercise with higher glycogen concentrations, an increase in glycogen use would be expected. Although muscle biopsies would be needed to rule out the role of muscle glycogen content on CHO oxidation in this study, both groups were fed a mixed diet (e.g., 55% CHO) on the day prior to testing in an effort to meet energy needs. Thus, differences in initial skeletal muscle glycogen content at the onset of exercise are unlikely to explain the lower CHO utilization seen between ECs and LCs during exercise. In fact, the similar exercise metabolic flexibility seen between groups would suggest comparable glycogen levels. Instead, prior work in chronic disease risk populations has suggested lower glycogen utilization during exercise in people with insulin resistance (Braun et al., 2004; Colberg et al., 1996; Goodpaster et al., 2002; Malin et al., 2013). As such, the shift towards carbohydrate in LCs may reflect preserved ability to utilize glycogen. These findings are somewhat supported by observations that greater fat oxidation during high‐intensity exercise is correlated with higher insulin‐stimulated NOGD. Thus, shifting towards CHO during exercise may contribute to decreases in endogenous CHO that collectively foster the disposition of glucose towards storage and/or oxidation during the insulin/fed state (Gilbertson et al., 2018). Whether aligning exercise by chronotype optimizes influences on insulin sensitivity awaits further investigation.

This study has limitations that warrant discussion. It is worth acknowledging that LCs often go to sleep later than ECs and this could have contributed to circadian misalignment (Eckel et al., 2015; Remchak et al., 2022; Simon et al., 2019; Yuan et al., 2021). Since all participants were asked to arrive at the lab at approximately 06.00–09.00 h, it is possible that this sleep restriction or social jetlag in people categorized as LC could have placed individuals in a quasi‐stressed state that activated greater reliance on CHO breakdown at fasting. However, all people exercised at the same time of day. This is worth considering as a strength since recent work highlights the time of day as an important consideration for molecular adaptations (Sato et al., 2022) as well as having potential implications for health (Remchak et al., 2021). Given our EC participants engaged in PA earlier in the day, future work ought to consider how exercise time of day aligns with chronotype for health benefit. Moreover, chronotype was defined using the 50th percentile as a proof‐of‐concept approach, as performed by us previously. Thus, we were unable to consider exact criteria for chronotype subgroups (e.g., definite morning, moderate morning, intermediate, moderate evening, definite evening). However, EC consisted of definite morning *n* = 2 and moderate morning *n* = 22, while LC was intermediate *n* = 20 and moderate evening *n* = 7, thereby providing confidence that we compared mostly early versus later chronotypes. ECs presented with higher fitness and engaged in more PA throughout the day. Whether raising fitness and/or LPA in LCs would induce comparable fat oxidation and/or NOGD remains to be seen. We did not directly assess sympathetic nervous system activity in the current study. Instead, we used HR as a surrogate and observed no difference between groups. Although we interpret this to suggest that neural activity was comparable, future work ought to consider approaches that assess sympathetic responses to exercise across chronotypes to enhance physiological understanding. Lastly, women in the present study were primarily post‐menopausal between both groups (EC *n* = 15 vs. LC *n* = 21) and remaining premenopausal women (EC *n* = 3 vs. LC *n* = 4) were reasonably matched, thereby suggesting modest, if any, influence on the findings.

In conclusion, ECs rely more on fat as an energy source during the fasted and moderate‐to‐high‐intensity exercise state compared to LCs in adults with metabolic syndrome. Interestingly, these findings occur independent of workload, suggesting that chronotype may be characterized by unique alterations in metabolism. Indeed, LC participants were more sedentary and had lower aerobic fitness than ECs. Interestingly, more LPA was positively related to metabolic insulin sensitivity, and higher fitness was related to increased exercise fat oxidation. Elevated exercise fat oxidation during high intensity was also associated with non‐oxidative glucose metabolism. Collectively, this work highlights and supports chronotype as a potential risk factor related to type 2 diabetes and CVD risk.

## AUTHOR CONTRIBUTIONS

Steven K. Malin conceptualized the work and design. Steven K. Malin, Mary‐Margaret E. Remchak, Anthony J. Smith, Tristan J. Ragland, Emily M. Heiston and Udeyvir Cheema contributed to data collection and/or analysis. Mary‐Margaret E. Remchak was mainly responsible for statistical analysis and data organization with support from Steven K. Malin, Anthony J. Smith, Emily M. Heiston and Tristan J. Ragland. Steven K. Malin and Mary‐Margaret E. Remchak primarily wrote the manuscript. In turn, all other authors edited the work. All authors have read and approved the final version of this manuscript and agree to be accountable for all aspects of the work in ensuring that questions related to the accuracy or integrity of any part of the work are appropriately investigated and resolved. All persons designated as authors qualify for authorship, and all those who qualify for authorship are listed.

## COMPETING INTERESTS

The authors report no conflict of interest.

## Supporting information

Statistical Summary Document

## Data Availability

These data have not been made publicly available. However, the corresponding author (S.K.M.) can provide further information on the data upon reasonable request.
